# Consumer Willingness to Pay for Food Defense and Food Hygiene in Japan: Cross-Sectional Study

**DOI:** 10.2196/43936

**Published:** 2023-10-23

**Authors:** Shinya Matsumoto, Yoshiyuki Kanagawa, Kiwamu Nagoshi, Takemi Akahane, Tomoaki Imamura, Manabu Akahane

**Affiliations:** 1 Department of Environmental Medicine and Public Health Faculty of Medicine Shimane University Izumo Japan; 2 Department of Public Health, Health Management and Policy Nara Medical University Kashihara Japan; 3 Department of Gastroenterology Nara Medical University Kashihara Japan; 4 Department of Health and Welfare Services National Institute of Public Health Wako Japan

**Keywords:** food defense, food hygiene, contingent valuation method, willingness, food, cost, awareness, food safety, questionnaire, Japan, prevention, food poisoning, safety

## Abstract

**Background:**

In Japan, incidents of falsified expiration dates on popular cookie brands and health hazards associated with frozen Chinese dumplings have raised food safety awareness. To prevent the intentional contamination of food by foreign substances, large food manufacturing companies have adopted the concept of food defense.

**Objective:**

The aim of this study was to assess people’s willingness to pay for food protection measures. In addition, the impact of participants’ personalities and considerations regarding their purchase choices on how much they were willing to pay when shopping for food and other products were measured.

**Methods:**

A questionnaire on willingness to pay for food hygiene and food defense was administered via a web survey and 1414 responses were included in the analysis. Univariate logistic regression analyses were performed with individuals willing and unwilling to pay additional costs as the objective variable and other questionnaire items as explanatory variables. A principal component analysis was performed on 12 questions regarding how much additional money people were willing to pay, and the principal component scores and other questions were examined for implications and other information.

**Results:**

Approximately one-third of the respondents stated that they were unwilling to pay additional costs and reported a willingness to consume delivery food even if it contained items that were not part of the original order. The first principal component reflected the extent to which people were willing to pay additional money, and if so, how much. This tendency existed even if the individual foods and amounts varied. The third principal component reflected the amount of extra money that people were willing to pay, which was determined by the amount people had to pay toward food safety measures. Those who answered “zero” were more likely to believe that consumers should not have to pay to ensure food safety. The second principal component reflected an axis separating food defense and food hygiene. Some items not directly related to food were correlated with this axis.

**Conclusions:**

In Japan, the concept of food hygiene is well-established and is generally taken for granted. In contrast, the concept of food defense is relatively new and has not yet fully penetrated the Japanese market. Our research shows that people who think that clothing brands provided added value to clothing products may have similar feelings about food defense. In addition, food hygiene efforts to prevent outbreaks of food poisoning are common in Japan and have been established as the basis of food safety. While food defense efforts are spreading, mainly in companies, it is presumed that they are valuable for the general public as supplementary measures to routine (or basic) food hygiene.

## Introduction

Terrorism can be carried out using various means, including food terrorism. Food terrorism is the act of intentionally contaminating food using poisonous substances to achieve political goals, causing harm to people and stirring up social unrest. Food defense represents an effort to prevent the intentional contamination of food [1-6]. Since the attacks of September 11, 2001, widespread attention has been directed to the danger of so-called food terrorism worldwide and building food defense [7]. Fortunately, no large-scale terrorist attacks using food have been reported to date; however, other issues plague food safety, such as cases of food counterfeiting or the falsification of expiration dates and a food’s place of origin. These issues have been reported as threats to food safety and security in Japan and elsewhere [8,9].

In addition to food hygiene measures, food defense measures are becoming more common to ensure food safety [10-13]. Food hygiene measures involve the monitoring of food and beverages to prevent foodborne health hazards. These measures include preventing spoilage by pathogenic microorganisms as well as contamination by harmful chemicals. To prevent spoilage and contamination, each stage of the food supply chain, such as production, transportation, packaging, preparation, and the sale of food and beverages, is undertaken with utmost care. In other words, the prevention of food poisoning enables maintaining a safe food supply chain while avoiding food poisoning. By contrast, food defense is a means to prevent health hazards caused by the intentional contamination of food and beverages with foreign substances [10,12,13]. Contamination in the food defense context is not accidental, such as that caused by human error, but rather involves incidents in which food or beverages are intentionally contaminated by foreign substances with malicious intent. Food defense measures are required to ensure food safety in all food aspects, both at the manufacturing level and at the retail and consumer levels.

Although not directly considered an act of food terrorism, intentional contamination or adulteration of food products with foreign substances has been reported in Japan and elsewhere [9]. In Japan, the health hazards caused by Chinese-made frozen dumplings in 2008 and the pesticide contamination of frozen foods manufactured in Japan in 2013 raised awareness for ensuring food safety during the food manufacturing process [10]. It has been approximately 10 years since efforts to prevent the intentional contamination of food were widely recognized as a food defense measure, especially by major food manufacturing companies in Japan [1].

To prevent health hazards caused by the intentional contamination of food, measures must be taken throughout the food supply chain. In other words, in addition to food defense measures adopted by food companies in food manufacturing and distribution and by retail stores and restaurants, citizens, as consumers, should also be aware of food safety. Therefore, in this study, a questionnaire survey was conducted to determine if and to what degree people are willing to pay for food safety and to identify the characteristics of people who perceive the risk of incidents that will threaten food in the future. In addition, the degree of willingness to pay to ensure food safety was surveyed. This study is the first to clarify whether and how much consumers are willing to pay for food safety.

## Methods

### Data Acquisition

This study was conducted as a cross-sectional survey using an internet panel survey company (Macromill Inc) and all participants were registered with the company. First, to recruit study participants, the survey company randomly sampled a list of registered participants between the ages of 15 and 79 years. Second, an email was sent to everyone on the list to inquire whether they were interested in participating in this study. Enrollment closed when the number of participants in each group by gender and age in 10-year increments reached the target sample size (103 people per group). Participants completed the survey via a web survey screen and received a small monetary reward upon survey completion. There were 1442 participants in the study; after data cleaning, 1414 participants were included in the analysis with 706 men and 708 women.

Although the Japanese population has a higher number of people in their 70s than in their 20s, all age/sex groups had an equal number of respondents in this study owing to the method used to enroll participants. We did not employ data augmentation methods because we used traditional statistical methods for the analysis. Regarding regions, there was a slightly higher number of respondents in the city prefectures of Kanto and Kansai than in other areas. The respondents’ incomes were categorized in ranges: the median household income of the respondents was 6-8 million yen (100 yen=US $0.9596), which is higher than the national median household income of 4.4 million yen.

### Questionnaire Items

Age, gender, weight, height, and region of residence were recorded when the participants first registered with the panel survey company.

In addition to items related to food safety, food hygiene, and food defense, the survey included questions about the participants’ personalities (self-evaluation) and lifestyles (what they considered important when purchasing electrical appliances and clothes).

The value of food hygiene and food defense was evaluated using the contingent valuation method (CVM). There were six questions each pertaining to the specific additional costs to be paid for food hygiene measures (Q27) and for food defense measures (Q28) for a total of 12 questions. The unit prices of several food products were provided as examples, and the respondents were asked how much extra they would be willing to pay for food hygiene or food defense measures to be incorporated. The relevant questions are presented in Textbox 1. The unit price is expressed in Japanese yen, and the exchange rate at the time of the survey, January 2021, was 100 yen=US $0.9596.

**Survey questions about additional costs (100 yen=US $0.9596).**

Q27. Food manufacturing companies in Japan have implemented food hygiene measures (ie, measures to prevent food poisoning), but these incur additional costs. How much extra would you be willing to pay for each of the following items owing to these measures?

(Please write “0” if you would not pay anything extra)

- 1000-yen lunch box
- 1000-yen juice
- 500-yen frozen food
- 500-yen side dish
- 500-yen snack
- 200-yen soft drink

Q28. Not all food manufacturing companies in Japan have implemented food defense measures (measures to prevent intentional contamination of food with foreign substances/drugs). Nevertheless, as these require additional expenditure, how much extra would you be willing to pay for each of the following items due to the measures?

(Please write “0” if you would not pay anything extra)

- 1000-yen lunch box
- 1000-yen juice
- 500-yen frozen food
- 500-yen side dish
- 500-yen snack
- 200-yen soft drink

### Data Analysis

We performed a distribution check on the payment amount for additional costs for food defense. As many people answered “0 yen” (not willing to pay), we performed comprehensive univariate logistic regression analyses for all combinations of the 12 objective variables and other questionnaire items as explanatory variables to analyze the characteristics of this subgroup. The univariate logistic regression analysis variable was evaluated using z-scores. By averaging the z-scores for the 12 questions (Textbox 1), we considered the strength of the relationship between the other questions and willingness to pay. The z-scores of the 12 logistic regression analyses, which changed the objective variable, were averaged and arranged in order of absolute values of the average of z-scores. The z-score is a statistic affected by sampling and therefore has small variation owing to sampling, whereas large fluctuations are unlikely. In this study, the responses to the 12 similar questions were converted to binary values and logistic regression was performed with the converted values as the objective variables. Given the slight variation in z-scores among the 12 logistic regression analyses, evaluating the 12 z-scores individually was not required and comparisons were made using mean values.

Since our questions concerned amounts of money, the data distribution was often skewed to the right (positive skewness). In the case of such a distribution, a transformation is often implemented to ensure that the distribution is closer to a normal distribution. As the Box-Cox transformation cannot be defined for 0, we added 1 to the transformation so that the distribution shape would follow a normal distribution. Since λ was estimated to be approximately 0 for each variable, we set λ=0 for all 12 variables. In other words, a logarithmic transformation was performed. Next, principal component analysis was performed on the 12 variables after the Box-Cox transformation and a principal component score was calculated for each respondent.

Univariate linear regression analyses were performed for all combinations with the top three principal component scores as the objective variables and all questionnaire items not used in the principal component analysis as the explanatory variables. Consequently, items that had strong relationships were identified. The relationships between each principal component score and the questionnaire items were examined.

### Ethical Considerations

This study was conducted with approval of the Ethics Committee of the National Institute of Public Health, Japan (approval number NIPH-IBRA#12302). All participants provided informed consent online for data collection and storage. Informed consent for participation in the study was obtained at the time of registration. In cases where participants were under the age of 18 years, a consent screen was displayed to obtain permission from their parents or guardians. The web-based questionnaire survey was conducted by an authorized research company compliant with personal information protection and we obtained anonymized data from the company.

## Results

The survey included questions regarding how much additional cost participants would pay for certain products. However, several respondents indicated they would pay many times more than the original unit price (double or more). The respondents who answered that they would pay more than 1.5 times the product’s unit price were considered outliers and excluded from the analysis. Therefore, responses for 1414 participants were included in the final analysis.

**Figure 1:**
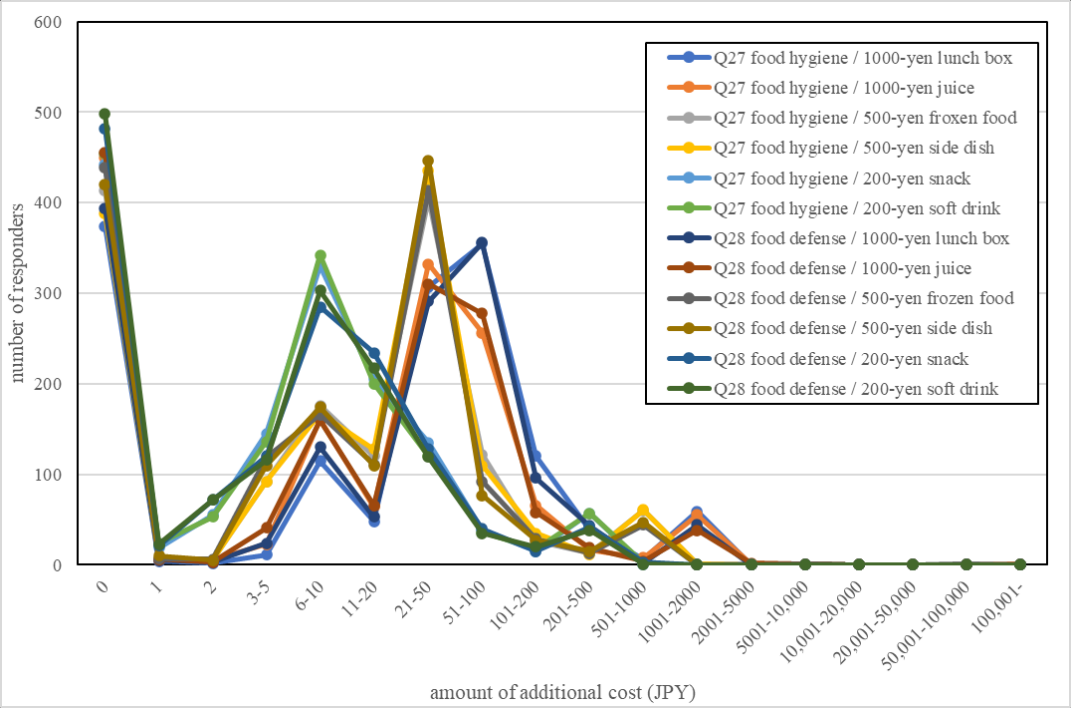
Distribution of the amount of additional costs (100 yen=US $0.9596). JPY: Japanese yen.

Figure 1 shows how much more money a respondent was willing to pay for each product according to the product type and unit price. Approximately 30% of respondents answered that they were not willing to pay any additional amount (0 yen) for each product. The most frequent value range, excluding 0 yen, was the same for food defense (Q28) and food hygiene (Q27): 51-100 yen for a 1000-yen lunch; 21-50 yen for a 1000-yen fruit juice drink, 500-yen frozen food, or 500-yen side dish; and 6-10 yen for a 200-yen snack and 200-yen soft drink.

Table 1 shows the median and mean additional costs for all respondents and for the respondents after data cleaning. Focusing on the average value after data cleaning, which is often used for CVM, the values were approximately 7% to 10% of the unit price. Comparing the same products, the amount of additional cost for food hygiene (Q27) was slightly higher than that for food defense (Q28).

**Table 1:** Summary of additional costs for the contingent valuation analysis (100 yen=US $0.9596).

Question		All responses			After data cleaning	
		Median additional cost (yen)	Mean additional cost (yen)	Median additional cost (yen)		Mean additional cost (yen)
**Q27: food hygiene**						
	1000-yen lunch box	50	121.80	50		101.60
	1000-yen juice	30	94.77	27.5		79.38
	500-yen frozen food	20	98.18	20		48.10
	500-yen side dish	20	64.58	20		49.28
	200-yen snack	10	24.47	10		20.71
	200-yen soft drink	10	24.12	10		20.21
**Q28: food defense**						
	1000-yen lunch box	50	103.8	50		87.85
	1000-yen juice	20	7715	20		69.91
	500-yen frozen food	10	108.6	10		41.57
	500-yen side dish	15	47.59	10		41.33
	200-yen snack	10	22.62	10		18.92
	200-yen soft drink	10	20.34	8		18.13

Table 2 shows the top 30 items in the analysis results. The first item was a direct question regarding paying additional costs; however, many questionnaire items that showed positive attitudes toward food defense and food hygiene were also present. Although they were not included in the top 30 items, people who were indifferent to food safety, such as those who would eat frozen food even if it had a strange smell, were also associated with paying 0 yen.

A principal component analysis was conducted on the 12 variables related to additional cost in the two major questions Q27 and Q28. The SD of the first principal component was greater than 5 and that of the second and third principal components exceeded 1.2 and were considered to be valid. Table 3 lists the factor loadings up to the third component. All the signs were the same for the first component. For the second component, Q27 and Q28 had differing positive and negative signs, respectively. In the third component, the loadings of the questions for expensive products were positive and those for cheap food products were negative.

**Table 2:** Top 30 items correlated with “0 yen” responses (unwilling to pay additional cost).

Question/statement	Average z-score
Please tell us about your thoughts on safety: I will pay additional fees to ensure safety	–7.423
When making a purchase, you take into consideration product labels (marks, etc) indicating that the product has been manufactured in a factory that adopts the following measures: food hygiene measures	–5.818
The following are important for you when buying food (here, the rating would indicate the degree of importance): safety	–5.373
Please tell us about yourself: I am cooperative	–5.202
You consider the following when purchasing food (here, the rating would indicate the degree of consideration given): expiry date/best-before date	–5.053
The following are important to prevent food poisoning when eating at home: cleanliness of the place where you are cooking	–5.023
The following are important for you when buying food (here, the rating would indicate the degree of importance): manufactured domestically	–4.841
When making a purchase, you take into consideration product labels (marks, etc) indicating that the product has been manufactured in a factory that adopts the following measures: food defense measures	–4.731
The following are important to prevent food poisoning when eating at home: expiry date	–4.622
Please tell us about yourself: I have a strong sense of morality	–4.561
The following are important to prevent food poisoning when eating at home: heat cooking	–4.505
You are worried the following will increase the risk of infection when eating at a restaurant amid the COVID-19 pandemic: condiments put on the table (containers with a lid)	–4.414
You practice the following often to maintain hygiene and cleanliness: wearing a mask	–4.400
The following are important for you when buying electrical appliances (here, the rating would indicate the degree of importance): safety	–4.384
You try to obtain new information on the following topics: domestic situation	–4.375
You are worried the following will increase the risk of infection when eating at a restaurant amid the COVID-19 pandemic: buffet-style layout	–4.326
When making a purchase, you take into consideration product labels (marks, etc) indicating that the product has been manufactured in a factory that adopts the following measures: allergen labeling	–4.274
You are worried the following will increase the risk of infection when eating at a restaurant amid the COVID-19 pandemic: drinks put on the table (pitchers with a lid)	–4.160
You try to obtain new information on the following topics: international situation	–4.150
When making a purchase, you take into consideration product labels (marks, etc) indicating that the product has been manufactured in a factory that adopts the following measures: labeling of foods for specified health use (FOSHU)	–4.145
Have you heard of the following terms? (Yes/No): food security and safety	4.107
You consider the following when purchasing food (here, the rating would indicate the degree of consideration given): ingredient labeling	–4.085
You practice the following often to maintain hygiene and cleanliness: hand washing	–4.020
Please tell us about yourself: I am diligent	–3.966
Considering that there have been several incidents of intentional contamination of food with foreign substances (pesticides, etc) in Japan, such incidents will recur in the future at the following places: food-related logistics facilities	–3.819
Please tell us about yourself: I have a strong sense of responsibility	–3.756
You are anxious about eating in the following style/places amid the COVID-19 pandemic (here, the rating would indicate the degree of anxiousness): buffet	–3.743
You worry about the following at international sports events held in the summer in Japan: heat stroke	–3.722
Personal income	3.719
You try to obtain new information on the following topics: environment	–3.677

**Table 3:** Factor loadings from principal component analysis.

Question		1st component	2nd component	3rd component
**Q27: food hygiene**				
	1000-yen^a^ lunch box	–0.33213	0.40084	0.29172
	1000-yen juice	–0.32377	0.41225	0.15839
	500-yen frozen food	–0.29709	0.24143	–0.18636
	500-yen side dish	–0.29346	0.25852	–0.12869
	200-yen snack	–0.23692	0.13195	–0.42668
	200-yen soft drink	–0.23624	0.13591	–0.41529
**Q28: food defense**				
	1000-yen lunch box	–0.33156	–0.19234	0.45951
	1000-yen juice	–0.32478	–0.20582	0.35734
	500-yen frozen food	–0.29400	–0.33029	–0.01778
	500-yen side dish	–0.28771	–0.32699	0.03124
	200-yen snack	–0.24059	–0.33121	–0.27116
	200-yen soft drink	–0.23670	–0.32140	–0.26522

^a^100 yen=US $0.9596.

Univariate linear regressions were conducted with the first to third principal components as objective variables and the remaining questionnaire items as explanatory variables. For the first principal component, there were 78 significant items (*P*≤.05). The top 30 items are listed in Table 4. For the first principal component, the strongest questionnaire item was “Please tell us about your thoughts on safety: I will pay additional fees to ensure safety.” The second strongest questionnaire item was “Please tell us about yourself: I am cooperative.” Other items also indicated that people who were interested in food hygiene and food defense were willing to pay additional costs. The items strongly correlated with the first principal component were considered to indicate the difference in attitudes toward food safety.

**Table 4:** Top 30 significant (*P*≤.05) questionnaire items in univariate linear regression for the first principal component.

Question/statement	*t* value (*df*=1412)
Please tell us about your thoughts on safety: I will pay additional fees to ensure safety	–7.4080
Please tell us about yourself: I am cooperative	–5.6810
When making a purchase, you take into consideration product labels (marks, etc) indicating that the product has been manufactured in a factory that adopts the following measures: food hygiene measures	–5.2480
The following are important for you when buying food: manufactured domestically	–5.0140
You are worried the following will increase the risk of infection when eating at a restaurant amid the COVID-19 pandemic: buffet-style layout	–4.9980
The following are important for you when buying food: safety	–4.9220
You are worried the following will increase the risk of infection when eating at a restaurant amid the COVID-19 pandemic: drinks put on the table (pitchers with a lid)	–4.8350
The following are important to prevent food poisoning when eating at home: expiry date	–4.8280
You practice the following often to maintain hygiene and cleanliness: wearing a mask	–4.7360
You are worried the following will increase the risk of infection when eating at a restaurant amid the COVID-19 pandemic: condiments put on the table (containers with a lid)	–4.7010
The following are important to prevent food poisoning when eating at home: heat cooking	–4.6800
You consider the following when purchasing food: expiry date/best-before date	–4.5550
When making a purchase, you take into consideration product labels (marks, etc) indicating that the product has been manufactured in a factory that adopts the following measures: allergen labeling	–4.4420
When making a purchase, you take into consideration product labels (marks, etc) indicating that the product has been manufactured in a factory that adopts the following measures: food defense measures	–4.3700
When making a purchase, you take into consideration product labels (marks, etc) indicating that the product has been manufactured in a factory that adopts the following measures: labeling of foods for specified health use (FOSHU)	–4.3230
You are anxious about eating in the following style/places amid the COVID-19 pandemic: buffet	–4.1600
You are worried the following will increase the risk of infection when eating at a restaurant amid the COVID-19 pandemic: disposable chopsticks (kept in a chopstick stand without packaging)	–4.1520
Have you heard of the following terms? (Yes/No): food security and safety	4.1430
The following are important to prevent food poisoning when eating at home: cleanliness of the place where you are cooking	–4.0640
Please tell us about yourself: I have a strong sense of morality	–3.9950
Have you heard of the following terms? (Yes/No): food terrorism	3.9630
The following are important for you when buying electrical appliances: safety	–3.8220
The following are important to prevent food poisoning when eating at home: best-before date	–3.7600
Age group	3.6820
If the frozen food you purchased is contaminated with foreign substances (metal, hair, etc), you…: dispose of it	–3.6720
Please tell us about yourself: I am diligent	–3.6160
The following are important for you when buying food: reputation (word of mouth)	–3.6090
The following are important for you when buying electrical appliances: reputation (word of mouth)	–3.5770
Personal income	3.5100
Age	3.4710

For the second principal component, there were 14 significant questionnaire items (*P*≤.05), which is considerably fewer than the number of significant items along the first principal component (Table 5). The strongest questionnaire item was “When you buy food, how important are the following: reputation (word of mouth),” followed by “When you buy clothes, how important are the following: price.” These are suggested to be related not only to food reputation but also to perspectives when purchasing items other than food.

**Table 5:** Significant (*P*≤.05) questionnaire items in univariate linear regression for the second principal component.

Question/statement	*t* value (*df*=1412)
The following are important for you when buying food: reputation (word of mouth)	–3.2050
The following are important for you when buying clothes: price	–3.0380
The following are important for you when buying food: price	–2.9940
Have you heard of the following terms? (Yes/No): food hygiene	2.6100
The following are important for you when buying food: brand	–2.4850
The frozen food you purchased has a bad (rotten, chemical, etc) smell. You…: dispose of it	–2.4530
You consider the following as “unhygienic” when eating at a restaurant: disposable chopsticks (kept in a chopstick stand without packaging)	2.4110
When making a purchase, you take into consideration product labels (marks, etc) indicating that the product has been manufactured in a factory that adopts the following measures: labeling of foods for specified health use (FOSHU)	–2.3840
When making a purchase, you take into consideration product labels (marks, etc) indicating that the product has been manufactured in a factory that adopts the following measures: food defense measures	–2.3780
The following are important for you when buying food: customer service/troubleshooting	–2.3310
The following are important for you when buying electrical appliances: brand	–2.0830
The following are important to prevent food poisoning when eating at home: order of cooking	–2.0710
When making a purchase, you take into consideration product labels (marks, etc) indicating that the product has been manufactured in a factory that adopts the following measures: food hygiene measures	–1.9830
The following are important to prevent food poisoning when eating at home: cold storage of ingredients	–1.9650

For the third principal component, there were 100 significant (*P*≤.05) items in univariate regression; the top 30 items are listed in Table 6. The strongest item was “age.” This analysis indicated that the items constituting respondents’ basic information were more relevant than the questionnaire items regarding food safety.

**Table 6:** Top 30 significant items (*P*≤.05) for the third principal component in univariate linear regression.

Question statement	*t* value (*df*=1412)
Age	6.2660
Age group	5.9030
Have child (Yes/No)	4.6800
Marital status	4.1900
You find extra item(s), which you had not selected, in the food parcel delivered to you. You…: eat it without worrying	–3.5670
You consider the following when purchasing food: place (ie, country) of production	3.4980
Please tell us about yourself: I am diligent	3.4700
You find extra item(s), which you had not selected, in the food parcel delivered to you. You…: contact the shop from where you had ordered it	3.4660
You consider the following when purchasing food: expiry date/best-before date	3.4190
Please tell us about your thoughts on safety: terror acts do not occur in Japan	3.3410
Please tell us about yourself: I am honest	3.1820
The following are important for you when buying food: safety	3.0710
There have been several instances of intentional contamination of food with foreign substances/drugs in the past, and implementing measures to prevent this is called “food defense.” Considering this, such measures should be adopted for food manufactured/provided at the following places: international political event venues (summits, etc)	3.0220
You are anxious about eating in the following style/places amid the COVID-19 pandemic: bars	2.9770
There have been several instances of intentional contamination of food with foreign substances/drugs in the past, and implementing measures to prevent this is called “food defense.” Considering this, such measures should be adopted for food manufactured/provided at the following places: food service chain stores (restaurants, etc)	2.9610
The frozen food you purchased has a bad (rotten, chemical, etc) smell. You…: contact the manufacturer	2.9070
There have been several instances of intentional contamination of food with foreign substances/drugs in the past, and implementing measures to prevent this is called “food defense.” Considering this, such measures should be adopted for food manufactured/provided at the following places: international sports event venues (Olympics, etc)	2.8990
You worry about the following at international sport events held in the summer in Japan: terror attack(s)	–2.8910
There have been several instances of intentional contamination of food with foreign substances/drugs in the past, and implementing measures to prevent this is called “food defense.” Considering this, such measures should be adopted for food manufactured/provided at the following places: food factories	2.7030
You are anxious about eating in the following style/places amid the COVID-19 pandemic: event venues (stores)	2.7010
Please tell us about yourself: I have a strong sense of responsibility	2.6900
The frozen food you purchased has a bad (rotten, chemical, etc) smell. You…: eat it without worrying	–2.5700
The frozen food you purchased has a bad (rotten, chemical, etc) smell. You…: contact the shop where you purchased it	2.5530
Please tell us about yourself: I have a strong sense of morality	2.4920
The following are important for you when buying food: manufactured domestically	2.4530
You practice the following often to maintain hygiene and cleanliness: wearing a mask	2.4410
You are anxious about eating in the following style/places amid the COVID-19 pandemic: home	–2.4120
When making a purchase, you take into consideration product labels (marks, etc) indicating that the product has been manufactured in a factory that adopts the following measures: food defense measures	2.3950
You practice the following often to maintain hygiene and cleanliness: cleaning	2.3640
The following are important for you when buying food: customer service/troubleshooting	2.1760

## Discussion

### Principal Results

In this study, a web-based survey was conducted in Japan to examine people’s willingness to pay for food safety and food defense. Approximately one-third of the respondents stated that they were unwilling to incur any additional costs. This subgroup also reported their willingness to consume food items included in their delivery even if those items were not part of their original order.

To analyze the additional amount paid, principal component analyses were performed. The first principal component reflected whether one is willing to pay extra and if so, how much. This propensity was maintained even if individual foods and amounts of money differed and therefore does not need to be discussed further. However, the second principal component reflected an axis indicating a difference in the importance of food defense and food hygiene among respondents.

### Food Hygiene and Food Defense in Japan

Individuals who expressed unwillingness to pay additional costs for food hygiene and food defense may perceive bearing the costs associated with ensuring food safety as the responsibility of businesses rather than that of consumers. The first principal component represented the amount paid, and many items strongly associated with this component were related to perceptions of safety. Some people may consider food safety as something that should be provided free of charge, whereas others are willing to pay for it.

Respondents who were unwilling to pay for additional costs were often those who would consume food included in their delivery even if it posed a risk of intentional harm. Therefore, it can be concluded that this type of respondent is less concerned about food safety. The third principal component reflected the influence of the original price. Few people indicated that they would pay a fixed amount higher than 0 yen, regardless of the product’s price. Those who claimed that the price would not affect their decision may also be considered in the 0 yen category; in other words, they have no intention of spending additional money. Consequently, it can be inferred that Japanese food is perceived as safe and trustworthy [2], and respondents with this perception do not intend to increase their expenditure.

Some individuals in Japan believe that investing in safety is unnecessary. For example, Japan’s tap water is inexpensive and suitable for drinking, and it is considered a safe country overall [14]. In 1970, Yamamoto (writing under the pseudonym Isaiah BenDasan) [15] astutely pointed out that “Japanese people think water and safety are free.” Despite this perspective, citizens do pay for water and contribute through taxes for police services. Owing to the relatively low cost and its integration with other needs, there is a high possibility that some citizens are unaware of the necessity of investing in safety.

In Japan, both food defense and food hygiene are crucial for ensuring safe and high-quality food. Food defense involves protecting food from intentional contamination, whereas food hygiene focuses on preventing unintentional contamination. Although government regulations strictly establish standards for food hygiene, guidelines for food defense are developed and published by a research team funded by the Ministry of Health, Labour, and Welfare’s scientific research grant [1,3]. In recent years, concerns have arisen regarding the intentional contamination of food products with foreign substances in Japan. Consequently, stronger food defense measures are required, and the industry is working toward addressing these issues and promoting greater transparency and traceability in the food supply chain.

Thorough measures to improve food safety in the food supply chain have become imperative recently [16,17]. As all factors at each stage of the food chain, from primary production to consumers, can affect food safety, necessary actions must be taken [18,19]. To mitigate the risk of food-related health hazards, both businesses involved in the food supply chain as well as consumers must adopt appropriate measures.

Overall, Japan has fostered a strong food safety culture and remains committed to ensuring citizen health and well-being through safe and nutritious food. However, the results of this survey indicate that some consumers may be unaware that maintaining Japan’s robust food safety culture, built over many years, may entail a financial burden.

The second principal component revealed an axis that distinguishes between food hygiene and food defense. The most important factor indicating this distinction was “When buying food, the following are important to you: reputation (word of mouth).” The second most important factor was “When buying clothes, the following are important to you: price.” The fifth most important factor was “When buying food, the following are important to you: brand.” Based on these results, the second most important factor, “clothing,” is not directly related to food, whereas the fifth factor, “brand,” emphasizes the value of intangible qualities. The value of a brand has also been demonstrated by Aaker [20] and Winters [21].

In the case of agricultural products, Koike et al [22] conducted an internet survey and found that 64.5% of consumers preferred domestically produced agricultural products, 35.2% had no specific preference, and only 0.3% preferred foreign-produced products. This indicates the existence of the “domestic myth” (an emotional belief that domestic production is superior) or the perception that food produced in Japan is inherently safe, particularly regarding agricultural products. In our study’s questionnaire, most respondents stated that domestic production is important when purchasing food because “domestic products are trusted by many people.” Additionally, individuals who value brands in contexts other than food, such as clothing, also support food defense. We asked respondents whether they considered “manufactured domestically” to be important when buying food, with 1.3% responding “strongly disagree,” 2.9% responding “disagree,” 10.9% responding “somewhat disagree,” 33.5% responding “somewhat agree,” 31.2% responding “agree,” and 20.2% responding “strongly agree.” This indicates that the “domestic myth” of domestic production is prevalent in the food industry in Japan as 84.9% of respondents answered “somewhat agree” or higher. Our findings suggest that food hygiene is well-managed and recognized in Japan, while food defense is considered an added value, similar to brands.

### Future Work

We intend to investigate causal relationships and other factors using analysis methods such as structural equation modeling to identify the type of consciousness the current result is based on.

### Limitations

Several limitations to this study must be acknowledged. First, the data were collected only from respondents registered with an internet panel survey company, which may have biased the sample. Older people who use computers and the internet may be healthier and more active than those who do not. However, panel surveys are increasingly widely used in questionnaires [5,23-26]. Second, respondents received a brief description of the survey via email and then chose whether to participate in the survey. Thus, there is a possibility of participant bias owing to their preferences. Third, we included an explanation of food defense in our survey because we thought that the concept of food defense was not well understood by the general public. In the future, we must clarify the difference between food defense and food hygiene and then ask the participants to respond. This may have affected some participants’ responses regarding food defense. Fourth, this was not a population-based study; therefore, the participants may not be representative of the general population in Japan. Fifth, participants received a small cash reward for their participation, which may have affected the randomness of the sample. However, the research firm minimized bias by using a large enrollment population to create the survey panel (the total number of people registered with the research firm was more than 1,300,000, accounting for approximately 1% of the Japanese population). Despite these limitations, internet questionnaires can be considered representative of the general public; therefore, we believe that the study results could represent the entire Japanese population to some extent.

### Conclusions

Approximately one-third of respondents said they were unwilling to incur additional costs for ensuring food safety. This subgroup also described that they would eat foods included in their delivery that they had not ordered. We performed a principal component analysis on the amount of additional money that people were willing to pay for food defense and food hygiene measures, and found an axis that indicated the divergent importance of food defense and food hygiene for consumers.

In Japan, food hygiene is taken for granted and food defense is considered of little significance by many people; it is only considered important by those who seek to gain peace of mind. Many companies are applying food defense efforts, but it is necessary to also educate consumers so that they understand the need for these measures.

## Abbreviations

CVM: contingent valuation method

## Data Availability

The data used in this study are available from the corresponding author upon reasonable request.
